# Telehealth at scale: the case for abandoning the paradigm of the “frequent flyer”

**Published:** 2012-06-15

**Authors:** Brian Hamilton

**Affiliations:** Message Dynamics Ltd, UK

**Keywords:** interactive voice response, (IVR), scale, low cost, self-management

## Abstract

The paradigm of the ‘pyramid of need’ and the relatively high per unit cost of telehealth has led to its use being targeted at supporting those ‘high-risk’ patients who it is widely believed account for a significant proportion of unplanned admissions. However, close examination of the frequency distribution of such admissions shows that the number of patients repeatedly admitted is low. This may explain why the dramatic reductions in rates of unplanned admissions reported by many telehealth projects have had little impact on the total number of unplanned admissions and thus healthcare costs.

Interactive Voice Response (IVR) reduces the costs of telehealth dramatically, is effective in capturing indicators of decreased well-being and, as the dialogue is symptom based, helps patients self-manage their condition. Low cost and the ubiquity of the telephone (mobile or landline) suggest that this technology is an economically and culturally acceptable means of screening large populations of patients.

